# Influence of Fever and Hospital-Acquired Infection on the Incidence of Delayed Neurological Deficit and Poor Outcome after Aneurysmal Subarachnoid Hemorrhage

**DOI:** 10.1155/2012/479865

**Published:** 2012-10-02

**Authors:** G. Logan Douds, Bi Tadzong, Akash D. Agarwal, Satish Krishnamurthy, Erik B. Lehman, Kevin M. Cockroft

**Affiliations:** ^1^Department of Neurosurgery, Penn State Milton S. Hershey Medical Center and College of Medicine, Hershey, PA 17033, USA; ^2^Penn State University College of Medicine, Hershey, PA 17033, USA; ^3^Department of Neurosurgery, SUNY Upstate Medical University, Syracuse, NY 13210, USA; ^4^Department of Public Health Sciences, Penn State Milton S. Hershey Medical Center, Hershey, PA 17033, USA

## Abstract

Although fever and infection have been implicated in the causation of delayed neurological deficits (DND) and poor outcome after aneurysmal subarachnoid hemorrhage (SAH), the relationship between these two often related events has not been extensively studied. We reviewed these events through of our retrospective database of patients with SAH. Multivariate logistic regression was used to determine independent predictors of DND and poor outcome. A total of 186 patients were analyzed. DND was noted in 76 patients (45%). Fever was recorded in 102 patients (55%); infection was noted in 87 patients (47%). A patient with one infection was more likely to experience DND compared to a patient with no infections (adjusted OR 3.73, 95% CI 1.62, 8.59). For those with more than two infections the likelihood of DND was even greater (adjusted OR 4.24, 95% CI 1.55, 11.56). Patients with 1-2 days of fever were less likely to have a favorable outcome when compared to their counterparts with no fever (adjusted OR 0.19, 95% CI 0.06, 0.62). This trend worsened as the number of days febrile increased. These data suggest that the presence of infection is associated with DND, but that fever may have a stronger independent association with overall outcome.

## 1. Introduction

Both clinical and laboratory studies have implicated inflammation in the causation of vasospasm and delayed neurological deterioration after aneurysmal subarachnoid hemorrhage (SAH) [1–12]. In addition, even mild hyperthermia has been shown to have deleterious effects on outcome in experimental models of ischemia [13–21]. In aneurysmal SAH patients specifically, fever and the systemic inflammatory response have been found to be associated with vasospasm and poor functional outcome [12, 22, 23]. Nosocomial infections have also been linked to poor outcome in this setting [24]. Despite this, the relative importance of fever, as compared to infection, remains controversial. In our study, we sought to examine the relationship between both fever and infection, and the incidence of delayed neurologic deficit (DND) as well as the impact of these events on overall outcome. We chose to use the term delayed neurologic deficit, rather than vasospasm, in order to emphasize the patient's clinical status and avoid the uncertainties surrounding the clinical significance of angiographic or transcranial Doppler (TCD) findings. Unlike previous studies, we attempted to include measures of fever and infection severity by including the number of days with fever and the number of infections as variables.

## 2. Materials and Methods

### 2.1. Subjects and Data Collection

We reviewed our retrospective neurovascular database for all patients admitted to the Penn State Milton S. Hershey Medical Center with aneurysmal SAH over a ten-year period. The review was approved by institutions IRB and no additional patient consent was required. All patients were either referred or directly admitted with a diagnosis of SAH. Patients found to have had traumatic SAH or nonaneurysmal SAH were excluded. In addition, in order to create a more homogenous data set, those patients that were found to have received neither surgical nor endovascular therapy were also excluded. Two sets of standardized forms were used to extract information from all the charts reviewed. Once all the raw data was collected from the charts, the information was entered into a Microsoft Access database.

From the database, in addition to basic demographic data, the following information was extracted: Hunt and Hess grade (H/H), Fisher grade, tobacco history, primary treatment modality (microsurgical or endovascular), incidence of TCD or angiographic vasospasm, incidence of fever, number of days febrile, incidence of infection, number of infections, incidence of delayed neurological deterioration, and Glasgow outcome score (GOS) upon hospital discharge.

Fever was defined as core temperature >38.3 degrees Celcius. If this elevated temperature occurred at any time in a 24-hour period this was considered a “febrile day.” For purposes of analysis, the number of days febrile was grouped into four categories (0, 1-2, 3–5, >5). An infection was defined as a clinical event marked by a positive culture (blood, urine, etc.) or positive imaging study consistent with infection (i.e., infiltrate on chest radiograph) that required treatment with antibiotics. The number of infections was also grouped into four categories (0, 1, 2, >2). TCD vasospasm was defined as a mean velocity of greater than 120 cm/sec in at least one vascular territory. Delayed neurological deterioration was defined as neurological worsening occurring at least 2 days after surgical/endovascular intervention and not attributable to any other cause such as hydrocephalus, rehemorrhage, seizure, or hyponatremia. Due to the inherent bias in selecting patients for cerebral angiography as well as the uncertain clinical significance of both angiographic vasospasm and elevated TCD velocities, we chose to use only the occurrence of a delayed neurological deterioration (DND) as a representation of “vasospasm.” GOS was dichotomized, with the favorable category, “excellent/good” representing GOS 4 and 5.

### 2.2. Statistical Analysis

A Pearson's chi-square test was used to test for a univariate association between each outcome variable and each categorical, but not ordinal, independent variable (covariate). A two-sample *t*-test was used as the univariate test for continuous variables. A Cochran-Armitage trend test was used as a univariate test for trend. Logistic regression was used to test the univariate association of age, gender, Hunt and Hess grade, Fisher score, tobacco use, treatment modality, incidence of fever, number of days with fever, number of days with fever category, incidence of infection, number of infections, and number of infections category on GOS category. DND was used as a covariate in the model to eliminate any confounding effect that it may have had on GOS category. Finally, we fit a multivariate logistic regression model for DND and GOS using the significant variables from our univariate analyses. Stepwise selection was used to add and remove variables to get the best fitting model. The inclusion/exclusion level of significance for this stepwise procedure was set at 0.15. These covariates were combined with the independent variable of interest in the final multivariate logistic regression model.

## 3. Results

A total of 615 patient records were reviewed based on presumptive diagnosis of SAH. Of these patients, 186 patients were actually found to have had aneurysmal SAH and to have received invasive treatment (Table 1). The median patient age was 54 years (range 8–88 years). Seventy-three percent (135 patients) were women and 65% (153 patients) were tobacco users. The median Hunt and Hess grade was 2.6 and the median Fisher grade was 3.1. The majority of patients were treated surgically (173, 74%). Only 20 patients (8.5%) had endovascular therapy (some patients were treated with both modalities, so the sum is not representative of total patients treated).

Fever, defined as core temperature >38.3, was recorded in 102 patients (55%); infection was noted in 87 patients (47%). The most common infections were urinary tract infection (UTI), pneumonia, line/catheter infection, and upper respiratory infection. There were numerous pathogens, but the most common ones were coagulase-negative *Staphylococcus*, *Candida albicans*, and *Escherichia coli*. There were no wound infections, empyemas, or intracranial abscesses in our study group. None of the patients met criteria for the definition of sepsis.

Delayed neurological deterioration (DND) suggestive of clinical vasospasm was seen in 76 patients (45%). One hundred twenty-seven patients (68%) underwent follow-up cerebral angiography at some point during their hospital stay, of these 65 studies (51%) demonstrated evidence of vessel narrowing consistent with vasospasm. All patients had daily TCD monitoring and TCD vasospasm (mean velocity > 120 cm-sec) were detected in 108 patients (46%) at some point in their course. A favorable outcome (GOS 4-5) was recorded in 122 patients (61%).

### 3.1. Univariate Analysis

We first tested for an association between DND and gender, tobacco use, treatment, fever, infection, and GOS category (Table 2). DND was significantly associated with GOS category, with an odds ratio of 0.39 (95% CI: 0.2, 0.78) indicating that a patient with DND was considerably less likely to be in the excellent/good GOS category when compared to a patient without DND. Fever was significantly associated with DND, with an odds ratio of 2.68 (95% CI: 1.42, 5.07), meaning that a patient with a fever was more than two and half times more likely to have DND than a patient without a fever. Infection was also significantly associated with DND with an odds ratio of 4.61 (95% CI: 2.4, 8.85). Gender, tobacco use, and treatment type were not significantly associated with DND. The mean age of patients with DND was not significantly different from those without (*P* = 0.51).

**Figure 1:**
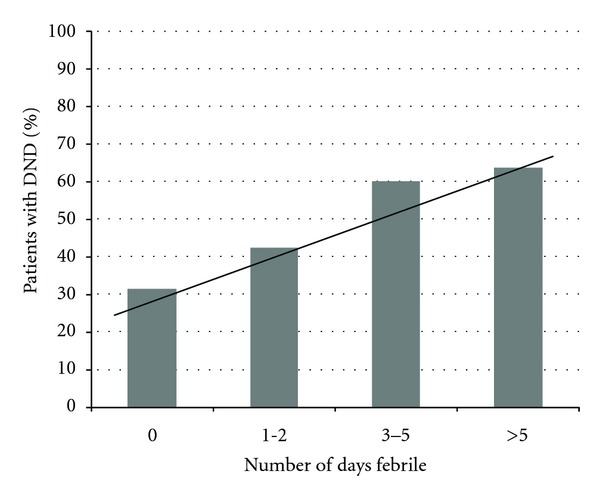
Rate of DND occurrence associated with increasing number of days febrile. A Cochran-Armitage trend test was used as a univariate test for trend (*P* < 0.01).

**Figure 2:**
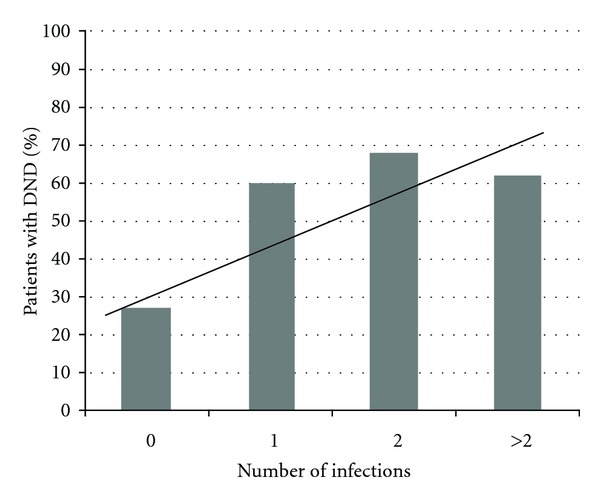
Rate of DND occurrence associated with increasing number of infections. A Cochran-Armitage trend test was used as a univariate test for trend (*P* < 0.01).

As expected, there was a significant positive trend in the number of patients with DND as Hunt and Hess grade increased (*P* < 0.01). Fisher score showed a similarly significant positive trend (*P* = 0.01). The number of days with fever category and number of infections category both demonstrated significant positive trends (Figures 1 and 2), with the percentage of patients developing DND increasing as the number of days with fever and the number of infections increased (*P* < 0.01, for both).

The association of age, gender, Hunt and Hess grade, Fisher score, tobacco use, treatment modality, incidence of fever, number of days with fever, number of days with fever category, incidence of infection, number of infections, and number of infections category with GOS category was analyzed (Table 3). Fever was negatively associated with GOS with an odds ratio of 0.12 (95% CI 0.05, 0.32, *P* < 0.01) indicating that a patient with fever was considerably less likely to be in the excellent/good GOS category when compared to a patient without fever. Infection was also significantly negatively associated with GOS with an odds ratio of 0.21 (95% CI 0.09, 0.46, *P* = 0.01). Number of days with fever (*P* < 0.01) and number of infections (*P* = 0.05) also demonstrated significant negative associations with GOS. Fisher grade and age were also correlated with a poor GOS. Gender, tobacco use, and treatment modality were not found to be significantly associated with GOS.

### 3.2. Multivariate Analysis

The variables that were initially included in the multivariable model for DND were number of days with fever, number of infections, Hunt and Hess grade, and Fisher score. Only number of infections remained in the model following stepwise selection (*P* < 0.01) (Table 4). A patient with one infection was more than three and one half times as likely to experience a DND as a patient with no infections (adjusted OR 3.73, 95% CI 1.62, 8.59). For those with more than two infections, the likelihood of a DND was over four times as great (adjusted OR 4.24, 95% CI 1.55, 11.56).

The multivariate model for GOS category (Table 5) initially included, DND, age, Fisher grade, number of days with fever category, and number of infections category. The final variables left in the model following stepwise selection were age (*P* < 0.01) and number of days with fever (*P* < 0.01); DND was then added back to this model as a covariate to get rid of any unwanted confounding effect. As expected, increasing age was associated with a lower chance of a favorable (GOS good/excellent) outcome (adjusted OR 0.96 95% CI 0.93, 0.98). However, for days with fever the effect was even more pronounced. Patients with one or two days of fever were about 20% as likely to have a favorable outcome as their counterparts with no fever (adjusted OR 0.19, 95% CI 0.06, 0.60). This likelihood of a favorable outcome worsened as the number of days febrile increased, with patients experiencing more than five days of fever being less than one-tenth as likely as those with no fevers to have a favorable outcome (adjusted OR 0.07, 95% CI 0.02, 0.21).

## 4. Discussion

Multiple clinical and laboratory studies have suggested that fever and/or a systemic inflammatory response may potentiate tissue damage in the setting of cerebral ischemia [13–19, 21, 25]. Certainly, SAH patients are frequently at risk for infectious complications [24, 26]. Such patients often have decreased levels of consciousness, which may be associated with an increased risk of aspiration pneumonia and prolonged intubation may increase the risk of ventilator-acquired pneumonia. Likewise central line infections, urinary tract infections, and CSF infections (cerebritis/meningitis) are not uncommon in this often critically ill patient population with multiple catheters and monitors in place for extended periods. However, noninfectious causes of fever are also frequent in this group [27], including atelectasis in the postoperative patient and DVT from prolonged bed rest. The presence of intracranial blood may in itself be sufficient to cause a febrile response.

A handful of studies have explored the influence of fever and the inflammatory response in subarachnoid hemorrhage patients [12, 22–24, 28, 29]. Previous case series have documented an association between fever and poor outcome in SAH as well as between fever, vasospasm, and increased mortality [22, 29, 30]. Infectious complications by themselves have also been shown to be associated with poor outcome [24]. However, a balanced comparison of the effects of fever and infection in the same population of aneurysmal SAH patients has not been undertaken. In addition, it is not clear whether a brief febrile episode or a single infection is sufficient to impact the incidence of DND or overall outcome.

In a prospective study by Olivera-Filho et al., 92 patients with nontraumatic SAH were analyzed [22]. The authors found an association between number of days febrile and poor outcome. This association was independent of hemorrhage severity. In the univariate analysis, infection alone was found to be a predictor of poor outcome, but the authors did not look specifically at the relationship between infection and vasospasm or delayed neurological deficit (DND). More recently, Dhar and Diringer performed a retrospective analysis of 276 nontraumatic SAH patients and evaluated them using a systemic inflammatory response syndrome (SIRS) score [28]. This value is derived from variables such as HR>90, RR>20, T>38C or <36C, WBC <4.000 or >12.000. The presence of SIRS had previously been shown to be associated with poor outcome after aneurysmal SAH [12]. Dhar and Diringer explored the SIRS concept in more detail by using graded scale of 0–4 to quantify the SIRS “burden.” Multivariate analysis found that SIRS burden was an independent predictor of vasospasm and poor outcome. The authors examined the specific infections of bacteremia, pneumonia, and UTI, but found no association between these infections and the presence of SIRS. In addition, these infections were not associated with the occurrence of vasospasm or poor outcome. Frontera and colleagues, on the other hand, looked primarily at infection and its impact on outcome after aneurysmal SAH [24]. Specifically, the impact of pneumonia, UTI, bloodstream infection (BSI), and meningitis/ventriculitis on outcome was studied. After adjustment for patient age, aneurysm size, and presenting clinical grade, the authors found both pneumonia and BSI to be significantly associated with death or severe disability.

Since previous studies have tended to focus on either fever/inflammatory response or specific-infection subtypes, we undertook the present analysis to determine which entity has the greatest degree of influence on the occurrence of DND and poor outcome. We chose to look at fever and infection specifically as they relate to delayed neurologic deficit and overall clinical outcome in the same patient population. Using number of days febrile and number of infections, we were able to demonstrate a trend toward increasing risk for DND and poor outcome as the presence of fever and infection increases. We recognized the numerous potentially confounding factors in assessing SAH patients and strove to account for those factors in our multivariate analysis. Fever, infection, the number of days with either, age and Hunt-Hess were all predictors of poor outcome. By conducting a multivariate regression analysis of these factors, the data revealed that the number of infections most strongly correlated with the presence of DND, while the number of days with fever showed a strong independent association with outcome. Although Hunt and Hess grade and Fisher grade were associated with DND in the univariate analysis, neither proved to be independent predictors of either DND or clinical outcome in the final multivariate models. Rather, patients with infections were approximately three to six times more likely to suffer a delayed neurologic deficit and patients with fever were approximately five to twelve times more likely to have a poor outcome.

These results suggest that both the presence of an infection and of fever itself (independent of an infection) may have an impact on the hospital course and eventual outcome of patients with aneurysmal SAH. This is the first report to implicate the number of infections as a related factor in the occurrence of DND. This further implies that aggressive treatment of only one of these entities in the absence of the other may be insufficient.

Obviously, this study has some limitations. Most notably, this is a retrospective series with a limited number of patients from a single institution. Due to the limited number of patients it was not possible to draw conclusions as to which particular infection or infections had more or less influence on outcome. With the present dataset it is also not possible to know with complete certainty, whether or not a particular febrile episode was directly related to a specific infection, or vice versa. In theory, a patient may have experienced a fever and a UTI, but the fever may not in fact have been due to the UTI. However, it is not clear that this impacts the overall findings of the study. Also of note, the majority of patients in this retrospective series underwent microsurgical clipping of their aneurysm rather than endovascular therapy; a trend that is essentially reversed in our current practice. Finally, we specifically analyzed only those patients who were actively treated for their SAH, so therefore many poor grade patients were not studied. As with any retrospective study of this nature, the results demonstrate association and do not confirm causation. There may be additional confounding factors not studied that influence the interaction of fever and infection with respect to DND and clinical outcome. It will be interesting to see if future studies assessing the impact of interventions for both fever and infection will have an impact on shifting outcome in a more favorable direction.

## 5. Conclusion

These data suggest that fever may be a strong independent predictor of poor overall clinical outcome after aneurysmal SAH. The presence of an infection alone was associated with the occurrence of DND after aneurysmal SAH and the more infections a patient experienced, the more likely they were to suffer a DND. Further study in a larger patient population may help better define the relationship between these factors and overall clinical outcome. In the meantime, these results suggest that aggressive ICU management of both fever and infection, even if not directly associated with each other, should remain of critical importance.

## Figures and Tables

**Table 1:** Baseline characteristics and outcome variables.

Number of patients	186
Age in years, mean ± SD	54.0 ± 14.2
Male (%)	51 (27)
Hunt and Hess grade, mean ± SD	2.6 ± 1.2
Fisher grade, mean ± SD	3.1 ± 1.0
Smoker (%)	153 (65)
Mean number of days febrile	2.8 ± 4.2
Number of days febrile category (%)	
0	84 (45)
1-2	35 (19)
3–5	31 (17)
>5	36 (19)
Mean number of infections	0.97 ± 1.4
Number of infections category (%)	
0	99 (53)
1	35 (19)
2	29 (16)
>2	23 (12)
Delayed neurological deficit (%)	76 (45)
GOS, excellent/good (%)	124 (67)

**Table 2:** Factors significantly associated with DND by univariate analysis.

Variable	Odds ratio	95% CI	*P* value
Fever	2.68	1.42, 5.07	<0.01
Infection	4.61	2.40, 8.85	<0.01
Favorable GOS	0.39	0.20, 0.78	<0.01

**Table 3:** Factors significantly associated with good outcome by univariate analysis.

Variable	Odds ratio	95% CI	*P* value
Increasing age	0.95	0.93, 0.98	<0.01
DND	0.37	0.18, 0.77	<0.01
Fever	0.12	0.05, 0.32	<0.01
Infection	0.21	0.09, 0.46	<0.01
Fisher grade*			0.02
2	0.58	0.06, 5.79	
3	0.20	0.02, 1.69	
4	0.13	0.02, 1.03	
Number of days febrile category^¥^			<0.01
1-2	0.19	0.06, 0.57	
3–5	0.13	0.04, 0.41	
>5	0.08	0.02, 0.23	
Number of infections category^Φ^			<0.01
1	0.29	0.11, 0.76	
2	0.24	0.08, 0.66	
>2	0.10	0.04, 0.32	

*Compared to Fisher grade 1.

^¥^Compared to a reference of zero days febrile.

^Φ^Compared to a reference of zero infections.

**Table 4:** Predictors of DND by multivariate analysis: impact of number of infections category.

Variable	Odds ratio	95% CI	*P* value
Number of infections category*			<0.01
1	3.73	1.62, 8.59	
2	6.20	2.38, 16.11	
>2	4.24	1.55, 11.56	

*Compared to a reference of zero infections.

**Table 5:** Predictors of good outcome* by multivariate analysis.

Variable	Odds ratio	95% CI	*P* value
Increasing age	0.95	0.93, 0.98	<0.01
DND	0.58	0.26, 1.29	<0.01
Number of days febrile category^¥^			<0.01
1-2	0.19	0.06, 0.62	
3–5	0.13	0.04, 0.41	
>5	0.08	0.02, 0.24	

*Good outcome = GOS 4 or 5

^¥^Compared to a reference of zero days febrile.
